# Organic Collaborative Research Partnership Building: Researchers, Needle and Syringe Program Providers, and People Who Inject Drugs

**DOI:** 10.3390/healthcare9111417

**Published:** 2021-10-21

**Authors:** Danielle Resiak, Elias Mpofu, Roderick Rothwell

**Affiliations:** 1 School of Health Sciences, Faculty of Medicine and Health, The University of Sydney, Sydney, NSW 2006, Australia; rod.rothwell@sydney.edu.au; 2 Rehabilitation and Health Services Department, University of North Texas, Denton, TX 76203, USA; 3 School of Human and Community Development, The University of the Witwatersrand, Johannesburg 2000, South Africa; 4 Family and Community Medicine, Meharry Medical College, Nashville, TN 37208, USA

**Keywords:** organic collaborative research partnership, harm reduction, NSP, PWID, community partnerships

## Abstract

(1) Background: People who inject drugs (PWID) and needle and syringe program (NSP) providers increasingly partner with researchers to explore harm reduction best practice. However, a paucity of research exists regarding how best to engage PWID and community NSP providers to generate the evidence for sustainable harm reduction services. (2) Aim: This study reports on our use of an organic community research partnership-building approach between researchers, NSP providers, and PWID in Canberra ACT, Australia. (3) Method: Survey participants included both PWID (n = 70) and NSP providers (n = 26) across primary (n = 2), secondary (n = 7), and outreach (n = 1) services in Canberra ACT. Applying an organic partnership-building strategy, we engaged with partners and adapted approaches according to information gained in the process of implementation. (4) Results: We found engaging in relationship building around partner priority activities created mutual understanding and trust premised in authenticity of the evolving partnership. Our organic approach, which included a partner audit of the research tools for relevance, resulted in high acceptance and enrolment into the research by NSP providers and PWID. Finally, we observed strong social capital building utilizing an organic approach for the sustainability of the partnership. (5) Conclusions: The results of this study provide evidence for the benefits of organic collaborative research partnership building with NSP providers and PWID for authentic service program implementation. Our approach to research partnership building resulted in strong relationships built on shared goals and objectives, mutual gains, and complementary expertise. We propose the wider use of organic approaches to developing collaborative research partnerships with NSP providers and PWID to enhance consumer responsiveness towards service provision.

## 1. Background

Collaborative research partnerships with consumers and health service providers facilitate the translation of research findings into policy development and implementation [1], closing the knowledge gap between health communities and researchers for improved health outcomes and quality of life (QOL) for community members [2,3]. However, the evidence is less clear as to how to build credible and trustworthy collaborative research partnerships with hidden communities, such as people who inject drugs (PWID) and their service providers. Globally, an estimated 11.3 million people inject drugs [4]. According to the United Nations Office on Drugs and Crime (UNODC) [4], PWID are at high risk of avoidable potential harms from substance use in the absence of evidence on how to support their safer use. The risks associated with substance use has further increased in recent years with fentanyl analogues serving as cheaper substitutes for heroin or as cutting agents [5]. Rapid sample techniques hold great promise for early interventions with PWID [6], and the benefits would likely multiply with collaborative research partnerships between researchers, PWID, and their NSP providers.

Research partnerships with PWID and NSP would provide credible, trustworthy, and dependable evidence for optimizing harm reduction practices for PWID, for whom abstinence may not be possible nor desired [7,8]. However, evidence is limited regarding collaborative research partnership strategies between NSP, PWID, and researchers with respect to evidence-based practices for improved health service access and outcomes for PWID.

### 1.1. Collaborative Research Partnerships: Their Nature and Significance

Collaborative research spans a broad range of approaches, such as “participatory research”, “participatory action research”, “action research”, “action science/inquiry”, “cooperative inquiry”, “participatory evaluation”, and “empowerment evaluation” [9]. These collaborative research approaches form a community of practice instrumental for knowledge development and management premised on collective learning and innovation [10]. The collaborating partners engage in all stages of research, from time, resource, and effort investment, to generate the evidence to answer significant clinical and programmatic questions. With collaborative research partnerships, the relationships between community partners and researchers can increase the data collection capacity, analysis, and interpretation; while enhancing program recruitment, sustainability, and extension [11]. However, we could not identify any studies on collaborative research partnership building between PWID and their NSP providers, despite recent advances in collaboration research, aimed to enhance practices with increased sensitivity to diverse population groups [12]. In response, we aimed to address this gap in methodological knowledge.

While academic research partners are uniquely positioned to provide the skills and expertise for rigorous and valid science, community stakeholders have the expertise to ensure the science is relevant and responsive to their community [13,14,15,16]. Theory and evidence informed community research partnership building is needed to guide best practice approaches with NSP service providers and PWID. Table 1 summarizes the essential components, processes, and outcomes to consider in collaborative research partnership building as applied to research partnerships with PWID and their NSP service providers (see also Oetzel et al. [17]).

### 1.2. Collaborative Research Partnership Approaches: Rationale

Community-based participatory research (CBPR) is a widely endorsed approach for its effectiveness among marginalized and vulnerable members of communities [18]. It is premised on trust and capacity building for community organizations and their clients. Organic approaches to collaborative research allow for frameworks of the partnerships to emerge naturally and in the context of typical research activities with would be partners, learning through implementing, and self-correcting from evidence gained in the process, while prioritizing partner interests [19]. For that reason, organic collaborative research partnership is a dynamic and evolving process with intuitive appeal in allowing the emerging partnership to be informed by the ongoing feedback it generates with implementation [20]. Organic approaches to partnership building have built-in flexibility to embrace the complexities of working within partner environments in ways that minimize the burdens of research participation with distributed efforts across typical activities of partner organizations and their clients. While researchers would be guided by a staged process to developing collaborative partnerships (see Table 2), they come to the partnership building with no preconceived notions about what is to occur nor timing of the specific events for the partnership building, appreciating the context for them.

### 1.3. The Present Study

Our study aims to provide evidence on the activities, process, and outcomes we engaged in organically, developing collaborative research partnerships with Australian PWID and their NSP providers. Our specific research questions were as follows:

1. What organic approach-emergent activities and processes characterize collaborative research partnership building with PWID and NSP providers?
2. What sustainable partnership outcomes result from implementing an organic approach to collaborative research building with PWID and NSP providers?

## 2. Method

### 2.1. Research Context

Harm reduction is a strategy aimed at minimizing harm to both individuals and the wider community from hazardous behaviors or practices that may not otherwise be completely eliminated [21]. NSP are an example of a harm reduction approach. Australia introduced NSP in 1986 [22] and now has in excess of 3000 NSP across primary, secondary, and outreach sites [23] (Kwon et al., 2012). Primary outlets are specifically established to provide the full range of NSP services, such as the provision of sterile injecting equipment, the collection of used injecting equipment, primary medical care in some instances, education, counselling, and referral services [24]. Secondary sites differ in that service provision at these outlets is one of many community health services provided. Secondary outlets are likely to include hospital emergency departments and community health centers [24]. Mobile and outreach services (as their name suggests) provide access to NSP to persons who are either hard to reach, are unable, or are unwilling to attend other outlets [24]. Mobile and outreach services allow PWID in remote or isolated regions access to NSP services [24]. In Australia, law enforcement is encouraged to work collaboratively with local NSP for safer use of substances by PWID [25,26]. These collaborations are at the core of community-based research, whereby much of the developments have occurred within the field of health [27].

### 2.2. Research Design and Procedure

The Ethics Review Committee (RPAH Zone) of the Sydney Local Health District approved the study (X17-0175 & HREC/17/RPAH/256). The conduct of this study at Australian Capital Territory (ACT) Health sites was authorized by the ACT Health Research Ethics and Governance Office (ETH.6.18.101E).

**Figure 1:**
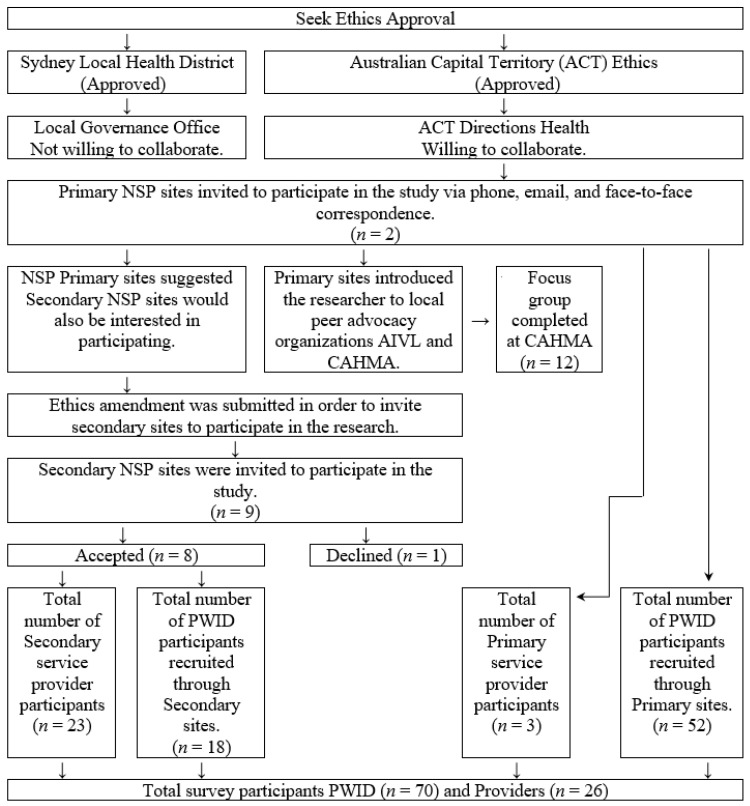
Flow chart of participant engagement. AIVL: The Australian Injecting and Illicit Drug Users League; CAHMA: Canberra Alliance for Harm Minimisation and Advocacy. Note: Source is author’s original work.

Our organic approach to research partnership building with NSP providers and their clients followed a participatory research approach [12], prioritizing local needs and perspectives to guide the research process [27]. We iteratively implemented the activities in Figure 1 at initial contact to inform subsequent online and in-person contacts.

Our initial partnership efforts involved contacting the Two primary NSP sites in ACT, advising them of the purpose and significance of the research partnership we were seeking, and discussing how the study could be carried out (i.e., two stages: surveys and then a focus group). The lead author followed up with meetings to share on our open-ended partnership-building approach with willing NSP partners.

During the study’s implementation, the lead author also met with representatives from both AIVL and CAHMA through primary NSP staff. AIVL is the Australian national peak organization who represent state and territory peer-based user organizations and issues of national relevance for people with lived experience of drug use. The Canberra Alliance for Harm Minimisation and Advocacy (CAHMA) is a peer-based alcohol and other drug service organization. This interaction led to additional collaborative partners and shared resources for the second phase of the study (the focus group discussion).

### 2.3. Participant Sampling

Our community partners were NSP service providers and PWID that collect their equipment from an included NSP. Altogether, we engaged a total of 10 NSP sites in the collaborative research partnership, resulting in a 26 NSP service provider and 70 PWID survey participation rate. Included NSP were existing NSP licensed to provide sterile injecting equipment to PWID, and the included PWID were NSP clients.

#### 2.3.1. NSP 

The NSP service provider staff that we engaged with were 18 years or above and had been employed or volunteered at an included NSP site for at least 1 year. We did not engage new NSP staff who were in training or new to the service, i.e., with less than 1 year of experience.

#### 2.3.2. PWID 

We engaged PWID through the NSP site they attended to collect sterile injecting equipment. Upon presentation at an NSP, PWID were asked by either the first-listed author or a staff member dispensing the sterile injecting equipment if they would like to participate in a research survey. Some PWID also informed peers in their network (other PWID) of the research partnership activities (the survey and focus group).

Eligible PWID were aged 18 years or above, who identified as a client of any of the NSP sites we engaged with for research collaboration. We offered the partner PWID a chocolate as a thank you for the time spent on the survey.

A summary of the number of service providers and users at each site is shown in Table 3.

### 2.4. Relationship Building

**Figure 2:**
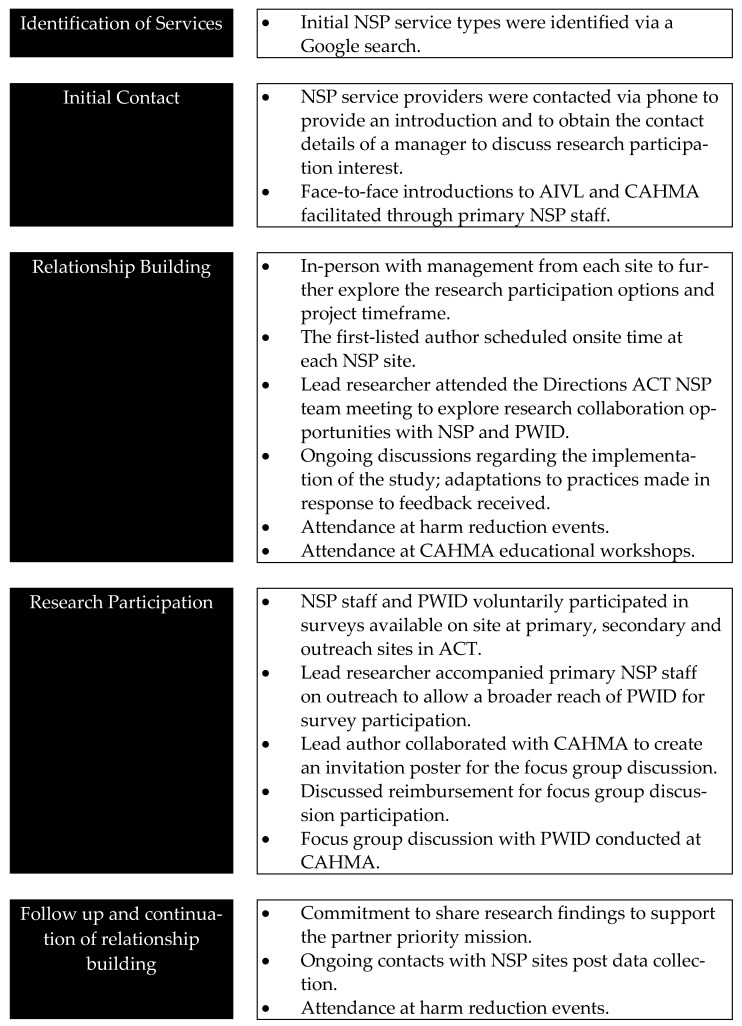
Process of relationship building. Note: Source is author’s original work.

Figure 2 provides a detailed description of the processes we engaged in to build relationships with potential research partners. True to the intent of organic approaches, we allowed partnership building to emerge as informed by and adapted according to experiences and lessons learnt in the process of implementation [19].

Our priority was trust building for partnership sustainability from the perspectives of credibility and transparency. Trust is a necessary condition of relationship building and is developed over time when effort and energy are invested into developing accessible and functional systems of communication [28]. Without trust, collaborations do not have a solid foundation on which to stand [28,29]. Partnerships that have gained trust are likely to result in long-term relationships bound by a shared understanding of the critical issues for the partnership, prioritizing partnership needs and outcomes [20].

Critical to relationship building was setting out the roles and responsibilities of the researchers, NSP service providers, and PWID. We discussed these at the initial meetings and while the lead author was on site at each of the NSP service locations. Table 4 presents the role and responsibility assignments for the research collaboration.

Demonstrating a shared vision and goals helped to strengthen the development of trust building between partners when genuine motives to improve consumer responsive services was observed. This occurred through time investment in activities above and beyond facilitating survey and focus group participation. For example, the lead author engaged in a number of peer-led educational workshops for PWID, attended harm reduction events, and volunteered time at NSP to engage with staff and PWID alike, in addition to accompanying NSP staff on outreach. Each community peer-led educational workshop covered a different topic relevant to improving the health and wellbeing of PWID, for example, overdose awareness and Naloxone training. The harm reduction events that were attended included the World Hepatitis Day Oration and the Hepatitis ACT World Hepatitis Day event. Participation in NSP-led community outreach activities signified to NSP providers and PWID the researcher’s investment in the health and wellbeing of those affected by substance dependency or addiction. Furthermore, alongside strengthening trust, being part of partner-led community activities allowed for a deeper understanding of the prospective participants realities.

## 3. Results and Discussion

Applying an organic collaborative approach to research partnership building resulted in strong relationships built on shared goals and objectives, mutual gains, and complementary expertise. These are hallmarks of successful researcher–practitioner collaborations [30]. Table 4 presents the role and responsibility assignments for the research collaboration and outcomes. We developed a strong trusting relationship with our PWID and NSP partners for win–win outcomes [31], utilizing organic partnership-building approaches. Organic approaches are best suited to developing mutuality in processes and outcomes as opposed to preordained approaches by external parties [32].

### 3.1. Relationship Building

Rapport development is integral to partnership development with marginalized populations such as PWID and their service providers [33]. With investment in rapport building, we acquired insider knowledge of the needs and priorities of PWID and NSP crucial for the success of a collaborative alliance due to an alignment of partnership interests and goals [34]. When community partners determine that a collaboration is closely aligned with their missions, strategies, and/or values, they tend to allocate more resources to relationship-building activities [34].

As evidence of differential relationship building, the first-listed author volunteered time at willing NSP sites, resulting in stronger rapport building as evidenced by the significantly higher participation rates among PWID (n = 52). Staff at the primary NSP sites expressed a personal investment in the outcomes of the study, stating a desire to continuously improve service provision for their clients. It was apparent that the NSP were invested in or connected to the research partnership development, increasing the likelihood of both their support for and participation in the research [35]. Secondary site participation was modest (n = 18), and there was little physical presence by our research team in their direct service activities. This suggests that we achieved a greater mission connection (improved consumer responsive NSP provision) with the primary sites as compared to the secondary sites. Whitehead, Hesselbein, and Austin [20] suggest that partnerships with a shared social purpose have an emotional connection, which is important for strong engagement. The shared alignment of goals also allows for continuous, iterative, and relational process in sustainable collaboration building [34].

We also observed that PWID and NSP provider engagement varied notably across primary and secondary site NSP locations. Australian secondary NSP outlets operate within existing services, such as sexual health centers, community health centers, or hospital emergency departments. While logical benefits for NSP provision exist in such settings, including improved access to supporting health and wellbeing services, diversity in secondary site activities meant a lower investment in NSP service engagement with clients and the research team. Primary NSP provider sites have a stronger partnership-building capacity for their clients PWID than programs with a broader focus, such as secondary NSP [36]. The diversity of activities at secondary NSP detracted from rapport building between the researchers and the PWID due to the asymmetrical relationship in priorities [37].

### 3.2. Trust Building

The need for trust building was apparent early on in our collaborative research partnership activities with NSP sites and their clients PWID. This was conducted in the knowledge that trust building is an evolving relational practice that is not static. It thrives on shared cultural norms, reciprocity expectations, and institutional arrangements, which shape social interactions [38]. Our trust-building strategy was focused on the establishment and maintenance of meaningful engagement with PWID and their NSP service providers in their own communities. Such partner-oriented trust building is critically important when working with marginalized population groups, who may already be distrustful of the intentions of academic research staff [38,39].

As part of the trust-building efforts, the first-listed author attended harm reduction events and peer-led educational workshops run by Canberra Alliance for Harm Minimisation and Advocacy (CAHMA) for a greater understanding of the context of PWID. Attendance at CAHMA’s educational workshops facilitated endorsed peer introductions that allowed the first-listed author to engage with prospective participants in shared tasks (Naloxone training and art activities), which encouraged a trusting relationship between PWID and the researchers that was nonjudgmental and free from stigma. Additionally, the first-listed author attended a guided tour of the Uniting Medically Supervised Injecting Centre (MSIC) to increase their understanding of how NSP can be extended beyond the provision of sterile injecting equipment, thus demonstrating a shared vision of support for improved healthcare for those affected by addiction or dependence. The tour included an introduction to the service, including its history, harm minimization approaches, an overview of substance use trends, client presentations, response to overdose, and the connection to wrap-around support services. Discussions of this experience with NSP service providers opened a dialogue in which shared visions were affirmed. Mutual trust and communication between PWID and NSP service providers is essential for improved health outcomes [40]. Distrust results in reduced service engagement [37] and partnership sustainability [40], especially with stigmatized populations [41].

### 3.3. Appropriateness of Methods and Interventions

We invited PWID to participate in a semi-structured focus group discussion based on the responses to surveys (completed by NSP service providers and PWID) on NSP service provision that we developed informed by the literature. This level of engagement provided credibility to the surveys, allowing for the inclusion of additional topics of importance to PWID. We invited participants to the focus group meeting using a poster that was co-designed and distributed by the lead author and CAHMA. This resulted in a total of 12 PWID participants for the focus group discussion. In [7], the authors highlight the importance of enhancing partnerships between researchers and PWID in public health research through community consultation. Moreover, the Australian Injecting and Illicit Drug Users League (AIVL) and CAHMA endorsed the survey materials for use with PWID and NSP.

### 3.4. Capacity Building and Project Resourcing

Our capacity building and project resourcing created social capital that resulted in sustainable partnerships that would not otherwise have been possible through independent efforts [28]. These social capital partnership-building activities were important for trust and reciprocity with our community partners [42]. We recognize that while material resources are important for collaborative activities over time, they are optimized with good social capital based on thoughtful relationship building. We also contributed to the operational capacity among NSP providers and PWID through not detracting from the provision of tangible resources like sterile needles and syringes, advice regarding safer injection practices, and referrals to peer support and advocacy.

### 3.5. Implications for Collaborative Research Partnership Building with NSP Providers and PWID

Needle and syringe programs are critical for harm reduction efforts globally, yet the process of forming collaborative research partnerships with NSP providers and PWID had not previously been reported. Collaborative research partnerships can provide evidence for improved consumer responsive services, informed by the priorities, and needs of both PWID and NSP service providers alike. Successful collaborative research partnership building enables “community-based/involved/collaborative/centered-research” [9]. We found that organic collaborative research partnership building with NSP providers and PWID was productive for building rapport, trust development, and nurturing this relationship. Our organic approach to collaborative partnership building had the advantage of allowing emergent health care needs to lead the direction and intensity of the partnership, without being constrained by a prior theory as to how the partnership would evolve. While theory-informed partnership development is important for benchmarking practices, context (e.g., cultural nuances) influences partnership development in unpredictable ways [11,17]. An organic approach, such as that which was implemented in this study, appears to have fared quite well for partnership building with PWID, their NSP service providers, and community advocates.

While researchers have the skills to carry out scientific enquiries, the results of such will be more meaningful if the process of enquiry includes the population bases in question. That is, NSP providers and PWID are best placed to ensure that the research aligns to the health needs of the community. Such collaborative research, utilizing complimentary expertise, is uniquely positioned to improve the health and wellbeing outcomes of PWID and the wider community of which they are a part with appropriately targeted and cost-effective NSP services.

The prevailing consensus is that collaboration is a journey rather than a destination [28] as it is an evolving partnership [12,20]. Our organic approach aimed at empowering the community partners [43], engaging them in formative activities for capacity building, sustainability, and program extension. Thus, our partnership-building approach committed to the co-creation of processes and tools for generating new knowledge and practices that would benefit PWID and their service providers.

### 3.6. Strengths and Limitations of the Study and Suggestions for Further Research

One in-built limitation with organic approaches is the indeterminacy of processes and outcomes. Since an organic collaborative partnership evolves based on practices and interactions with the community partners in the context of their typical activities, new issues come up calling for further partnership adaptation. There is not, as yet, a saturation algorithm for determining when to end partnership development activities. We also acknowledge the limitation that our organic collaborative research partnership-building process included a Canberra ACT population of NSP service providers and their client PWID. The findings may be different across other regions and jurisdictions. The processes and outcomes may vary widely depending on the partners involved and the study context, which would limit the direct comparison of collaborative research partnership outcomes. Nonetheless, we believe our study provides a basis for future organic approach partnership-building activities with PWID and NSP, embracing the complexities of organic approaches.

## 4. Conclusions

The results of this study provide evidence as to the benefit of organic collaborative research partnership building with NSP service providers and PWID for authentic service program implementation. Through our organic collaborative partnership-building approach, we built strong relationships with PWID and NSP through prioritizing partner missions and goals, volunteered time, capacity building, and appropriate methods of engagement. The collaborative effort resulted in shared social capital and material resources, which increased access to the partner NSP providers and their client PWID for the mixed methods studies to follow. Our major innovation was to demonstrate a bottom-up approach to building research partnerships with an often-hidden population of PWID and their NSP providers, without imposing preordained participant recruitment and engagement procedures on them. In doing so, we provided a significant contribution to participation action research theory based on organic approaches. We propose the wider use of organic collaborative partnership-building approaches, as their flexibility to adapt to evolving partnership dynamics allows for stronger partnership bonds to be formed, trust to be gained, and enhanced research translation into practice. The downstream social and economic benefits to the design and implementation of NSP services for PWID should include greater service access at a lower cost, with services customized to the needs of PWID and their service providers.

## Figures and Tables

**Table 1:** Components of the collaborative community—academic partnership.

Components	Descriptions and Process	Outcomes
NSP Context	Partnership capacity Final approval	Knowledge of NSP site capacity and barriers to service provision
Partnership Structures	Shared control of resources	Understanding of NSP structure types
Partnership Structural Values	Bridging social capital Alignment with CBPR partnership focus principles Core values	Alignment of shared goals Partnership commitment Improved client health and wellbeing through service provision and referral
Relationships	Participation Cooperation Respect Trust Participatory decision making Leadership Resource management	Personal capacity building Agency capacity building Sustainability of partnership
Intervention	Community involvement in data collection (survey and focus group) Community involvement in dissemination Partnership synergy	Service engagement Health outcomes Community health improvement

Note: Source is author’s original work.

**Table 2:** Community-based participatory research stages.

CBPR Stage	Elements Included in the Stage
Stage One	Defining the community Engaging the community Community needs assessment Identifying research question
Stage Two	Design/hypothesis testing Roles and responsibilities
Stage Three	Analysis Interpretation and results Dissemination and action

Note: Source is author’s original work.

**Table 3:** Summary of survey participants by site.

ACT Research Site	Site Type	Number of Service Provider Participants	Number of Service User Participants
City Health Centre	Primary	3	35
Phillip Health Centre	Primary	0	14
Aids Action Council	Secondary	7	4
Alcohol & Drug Service	Secondary	5	3
Belconnen Community Health Centre	Secondary	1	0
Gungahlin Community Health Centre	Secondary	0	1
Tuggeranong Community Health Centre	Secondary	6	0
Hepatitis ACT	Secondary	4	1
CAHMA	Secondary	0	9
Outreach	Outreach	0	3
Totals	-	26	70

Note: Source is author’s original work.

**Table 4:** Role and responsibility assignments and outcomes.

Stakeholder	Roles and Responsibilities	Outcomes
Researchers	Submit research project for ethics approval. Identify NSP for community-based partnership research. Co-design and facilitate the research project. Engage in partnership-building activities.	Ethics approval granted. There were a total of 11 NSP sites identified, 10 of which participated (2 Primary; 8 secondary). Five research flyers were displayed across sites to assist with recruitment. Strengthened partnerships resulted in greater collaborative participation. NSP and PWID centered research questions and procedures used.
Needle and Syringe Program (NSP) providers	Identify point of contact at each site for the community-based participatory research. If willing, participate in the service provider survey. Help to foster trust-building activities between researchers, NSP providers, and clients (PWID).	Contacts for each site were established and the lead author was invited to attend the Directions Health NSP team meeting to discuss the research, and allow potential collaborators’ questions to be answered and feedback to be provided. Confidentiality and anonymity in data collection and reporting resolved. A total of 26 NSP provider surveys were completed. NSP service providers informed PWID attending the NSP of the survey and focus group opportunity, which provided a trusted endorsement. Facilitating shared outreach activities and additional connections with prospective participants fostered trusting relationships.
People Who Inject Drugs (PWID)	If willing, share experience of NSP through a survey and/or a focus group discussion. If desired, tell networked prospective participants about the emerging collaborative research partnership. Raise any comments or concerns regarding the emerging collaborative research partnership. Help to foster trust-building activities between researchers and other PWID.	Confidentiality and anonymity issues in data collection and reporting resolved. A total of 70 surveys were completed by PWID at their NSP of choice. A trusted safe space for the focus group discussion was identified and confirmed. The focus group discussion meeting attracted a total of 12 participants (the maximum number that could be facilitated). Peer organization members alongside PWID who took part in the survey informed peers of the survey and focus group participation opportunities, which expanded the reach of the research.

Note: Source is author’s original work.

## Data Availability

Data are not publicly available due to the confidential nature of the information.
